# Enhancing the Responsivity of Uncooled Infrared Detectors Using Plasmonics for High-Performance Infrared Spectroscopy

**DOI:** 10.3390/s17040908

**Published:** 2017-04-20

**Authors:** Amr Shebl Ahmed, Hye Jin Kim, Jinsik Kim, Kyo Seon Hwang, Seonghwan Kim

**Affiliations:** 1 Department of Mechanical and Manufacturing Engineering, University of Calgary, Calgary, AB T2N 1N4, Canada; shebl99@gmail.com; 2 Center for BioMicrosystems, Korea Institute of Science and Technology, Seoul 02792, Korea; T12555@kist.re.kr; 3 Department of Medical Biotechnology, Dongguk University, Seoul 10326, Korea; lookup2@dongguk.edu; 4 Department of Clinical Pharmacology and Therapeutics, Kyung Hee University, Seoul 02447, Korea

**Keywords:** plasmonics, infrared detector, MEMS, gas sensing

## Abstract

A lead zirconate titanate (PZT;Pb(Zr_0.5_2Ti_0.48_)O_3_) layer embedded infrared (IR) detector decorated with wavelength-selective plasmonic crystals has been investigated for high-performance non-dispersive infrared (NDIR) spectroscopy. A plasmonic IR detector with an enhanced IR absorption band has been designed based on numerical simulations, fabricated by conventional microfabrication techniques, and characterized with a broadly tunable quantum cascade laser. The enhanced responsivity of the plasmonic IR detector at specific wavelength band has improved the performance of NDIR spectroscopy and pushed the limit of detection (LOD) by an order of magnitude. In this paper, a 13-fold enhancement in the LOD of a methane gas sensing using NDIR spectroscopy is demonstrated with the plasmonic IR detector.

## 1. Introduction

IR radiation detectors can be classified into two major categories: photon detectors (also known as quantum detectors) and thermal detectors. Photon detectors mainly depend on the quantum interaction between incoming photons and electrons [1]. Incoming photons get absorbed by electrons in the sensing material, changing their electronic energy distribution then resulting in electrical output signal of photon detectors. Thus, photon detectors generally demonstrate high performance with a very fast response. However, since thermal noise in photon detectors increases exponentially as a function of temperature, *T*, high signal-to-noise ratio (SNR) of photon detectors can be achieved by cryogenic cooling. This cooling requirement makes photon detectors more bulky, heavy, costly, and difficult to miniaturize. On the other hand, thermal detectors (pyroelectric [2], thermoelectric [3], thermoresistive [4], and thermomechanical [5] sensors), relying on IR absorption induced temperature change, can operate at or even above room temperature without cryogenic cooling. However, these uncooled IR detectors generally demonstrate low performance with a slow response compared to photon detectors. Therefore, there is very high demand to develop miniaturized, cost-effective, uncooled IR detectors with excellent performance [6]. Although thermal detectors can also be cooled to enhance the performance, cryogenic cooling is not as effective as in photon detectors since thermal noise in thermal detectors increases as $\sqrt{T}$. The noise level or detection limit of these two types of IR detectors as a function of IR wavelength, background temperature, and detector temperature are well described in previous literatures [7,8].

With the rapid advancement of microfabrication techniques, high-performance uncooled IR detectors have been extensively explored and developed in the form of microelectromechanical systems (MEMS) over the last two decades. For example, bimetallic microcantilevers with absorptive coatings have been exploited as ultrasensitive thermal sensors for uncooled IR detection and imaging [5,9,10,11]. However, these microcantilever structure-based IR detectors are very susceptible to intrinsic mechanical stresses, external vibrations, and ambient temperature fluctuations. To overcome these problems, several innovative designs have been investigated and reported [12,13,14,15]. Here, we report on the design, fabrication, and characterization of a PZT micromembrane-based IR detector decorated with a wavelength-selective plasmonic crystal structure and demonstrate its application in NDIR spectroscopy. Plasmonics, the use of localized plasmons and surface plasmon-polaritons (SPPs), has been widely studied in the visible regime and now rapidly extending to the mid IR regime [16]. SPPs are surface confined waves that propagate at the interface between a conductor and a dielectric material [17]. Although the significant losses associated with their propagation hindered their wide use for many applications [18,19], they can be exploited to enhance the performance of IR detectors by enhancing the absorption of IR radiation. Thus, SPPs have been recently employed to enhance the responsivity of IR detectors [3,20,21,22].

NDIR spectroscopy is one of the most widely-used optical gas sensing techniques [23]. It is the first optical gas sensing technique developed by Luft in 1943 [24]. In the previous literatures, many techniques of enhancing the LOD of NDIR gas spectroscopy have been reported by introducing optimal designs of the gas flow cell for a longer optical path length [23]. However, in this paper, we demonstrate another strategy for an order of magnitude enhancement in the LOD of NDIR gas sensing system by enhancing the responsivity of the pyroelectric thermal IR detector using a plasmonic crystal structure.

## 2. Design and Simulations

**Figure 1:**
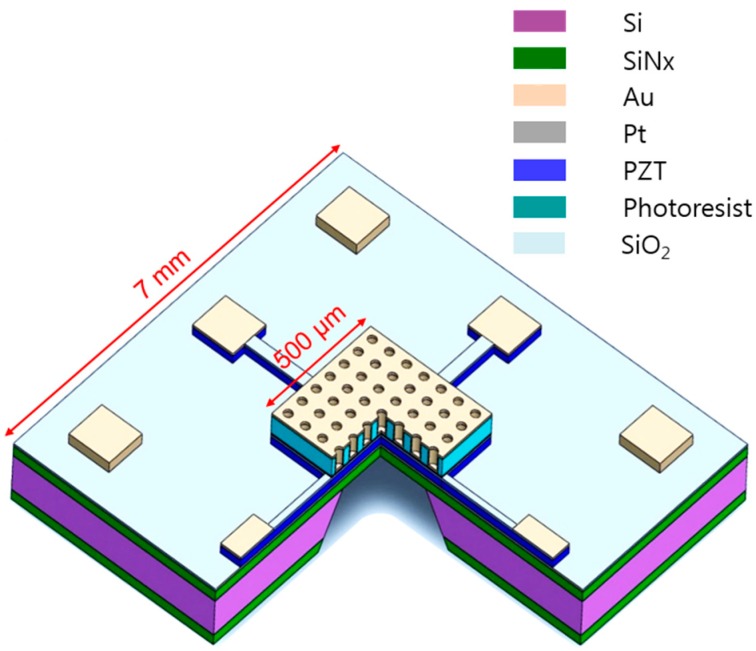
A schematic diagram of the pyroelectric IR detector with a plasmonic crystal structure. The cross-sectional part shows the micro membrane structure of the detector.

**Figure 2:**
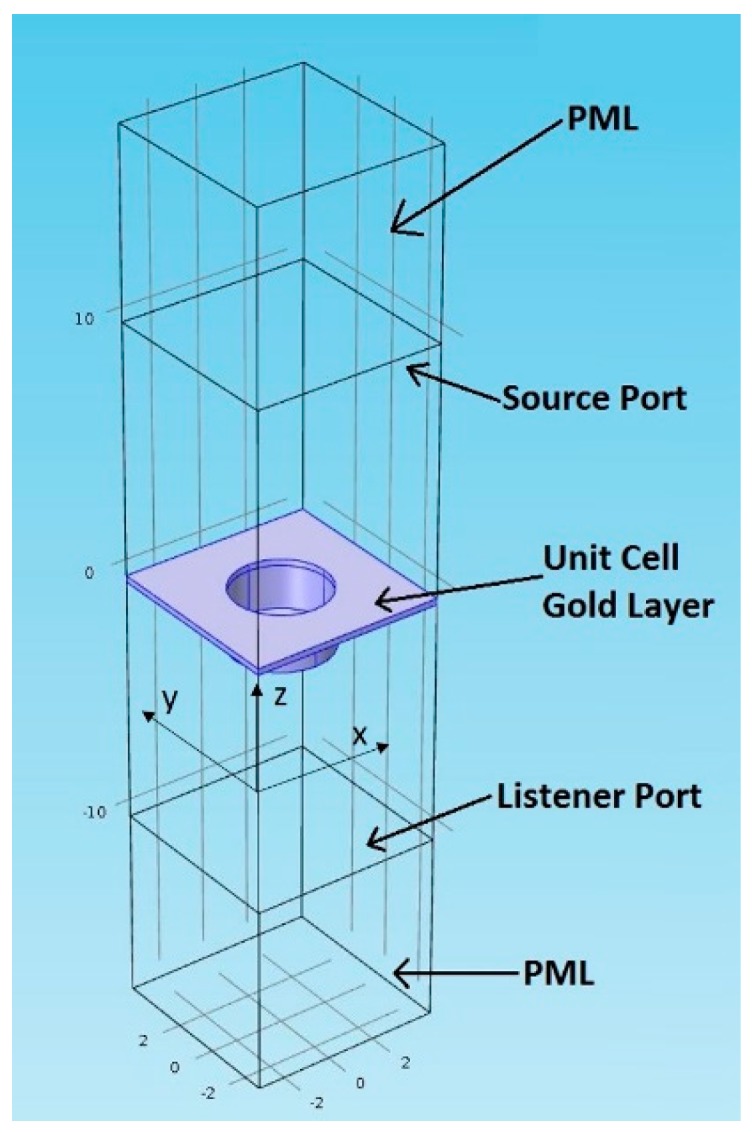
A schematic diagram of the numerical simulation model. Only a unit cell of the plasmonic crystal structure is simulated considering the periodicity of the two dimensional array.

Figure 1 represents a schematic diagram of the PZT layer embedded, pyroelectric thermal IR detector with a plasmonic crystal structure. The IR detector is decorated with a gold-coated two-dimensional array of holes. When the IR radiation is selectively absorbed by the plasmonic crystal structure, the temperature of the IR detector increases which results in a voltage change between top and bottom platinum electrodes. There are four parameters that define the dimensions of the two dimensional plasmonic crystal structure: the array pitch *p*, the gold layer thickness *t*, the hole radius *r*, and the hole depth *d*. The combination of these parameters determines the wavelength at which SPPs are excited and IR absorption is enhanced [3]. Finite element simulations are performed using commercially available software, COMSOL^TM^ RF Module, to optimize the dimensions of the two-dimensional plasmonic crystal structure for maximum absorption around the wavelength λ = 7.7 µm which has multiple characteristic IR absorption peaks of methane [25]. These absorption peaks are targeted specifically since they are characteristic peaks to methane over other hydrocarbon molecules. In addition, this wavelength range is selected to satisfy the minimum feature requirements of the structure achievable by conventional photolithography. A schematic diagram of the model we used for the numerical simulations is shown in Figure 2. Because the plasmonic crystal structure is a periodic structure, only a unit cell of the structure is simulated based on the assumption that the gold structure is infinitely wide. The unit cell shown in Figure 2 represents the gold layer whereas all other domains are considered to be air. The optical properties of the gold layer are taken from [26]. In all simulations, the thickness of the gold layer is at least four times the skin depth (25 nm) of gold layer in the mid IR range [27]. Subsequently, the underlying layers are not simulated. Above the gold layer, we have a source port boundary condition. Throughout the numerical study, the IR radiation is linearly polarized with zero angle of incidence. Below the gold layer, a listener port boundary condition is applied. The reflection coefficient, R, and the transmission coefficient, T, are numerically calculated and the absorption coefficient A is determined be A = 1 − T-R. The domains above the source port and below the listener port are perfectly matched layers (PMLs) to model open boundaries. The periodicity of the structure is accounted for by applying Floquet periodic boundary conditions on each two opposite sides of the unit cell. The size of the mesh elements is constrained to be smaller than λ/10 to make sure the wavelength is well resolved.

The optimized dimensions for our application are determined to be *p* = 7.65 µm, *t* = 100 nm, *r* = 1.5 µm, and *d* = 2 µm. The details of the results of the optimization process using numerical simulations are provided in the appendix.

The thermal time constant of the designed sensor is calculated using ANSYS^TM^ finite element simulation software. The properties of the materials used in the simulation are shown in Table 1. A constant power of 1 mW is applied to the surface of the sensor and the average temperature of the pyroelectric layer is calculated over time. The thermal time constant of the sensor is found to be 7 ms.

## 3. Fabrication

**Figure 3:**
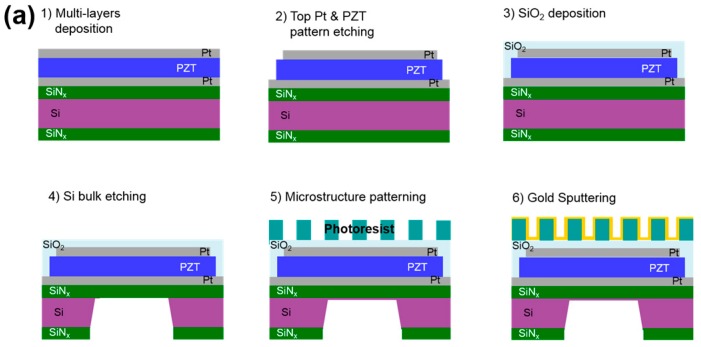

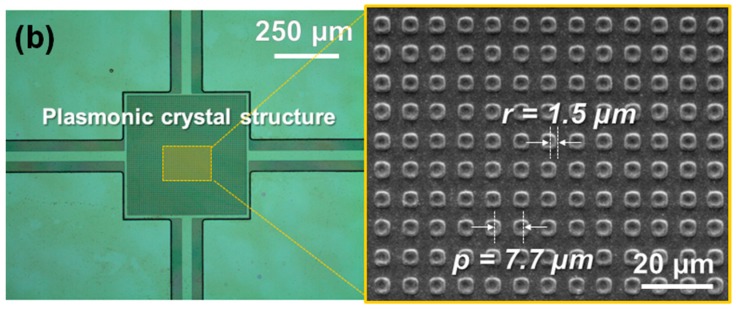
(**a**) Schematic illustration of the fabrication processes of the pyroelectric IR detector with a plasmonic crystal structure; (**b**) An optical microscopy image of the fabricated plasmonic crystal structure along with a magnified scanning electron microscopy image.

The pyroelectric IR detector in micro membrane shape is fabricated following the processes shown in Figure 3a. The IR detector is a 500 μm square-type membrane and the thickness is of about 3.45 μm [28]. First, the multilayer consisting of the Pt/PZT/Pt/Ta/SiNx/Si/SiNx is prepared for fabrication of the pyroelectric IR detector (Step 1). The SiNx with a thickness of 1.0 μm is deposited on the four-inch Si wafer by a low pressure chemical vapor deposition (LPCVD) method and Pt/PZT/Pt/Ta multilayer is deposited on the SiNx layer. The PZT layer is coated by a sol-gel method. The PZT solution is prepared using lead acetate trihydrate, zirconium propoxide, titanium isopropoxide and 1,3 propandiol and acetylacetone and deposited by spin-coating at 3000 rpm for 30 s and subsequently baked at 400 °C for 5 min and 650 °C for each layer [29]. The top and bottom Pt layers and the Ta layer under the bottom Pt layer are deposited by a RF sputtering. Thickness of the PZT layer and up and bottom side Pt layers are 2.0, 0.15, and 0.1 μm respectively. Also, thickness of the Ta layer is 0.03 μm. After formation of the multilayer, these layers are etched and the SiO_2_ layer with a thickness of 0.2 μm is deposited for passivation (Step 2 to 3). Then, the Si layer underlying the pyroelectric detector is etched out to thermally isolate the IR detector and enhance its responsivity (Step 4). To fabricate the plasmonic crystal structure at the upper side of the pyroelectric detector, about 2 μm thick photoresist polymer AZ GXR 601 46 cP (AZ Electronic Materials, Darmstadt, Germany) is coated by controlling the rpm of a spin-coater. Then, the photoresist polymer layer is patterned using the photolithography process to fabricate a micro-hole pattern with pitch of about 7.7 μm and radius of about 1.5 μm (Step 5). Finally, a 100-nm layer of gold is sputtered with a shadow mask to take the final shape of the plasmonic crystal structure (Step 6). Although robust pattern could be generated with thick silicon dioxide layer, the photoresist is chosen here due to the limitation of our fabrication facility. Figure 3b shows an optical microscopy image of the IR detector decorated with a positive photoresist and a magnified scanning electron microscopy (SEM) image of the IR detector with a gold plasmonic crystal structure. The 500-µm square-type membrane detector has 3364 holes with *p* = 7.7 µm, *t* = 100 nm, *r* = 1.5 µm, and *d* = 2 µm. The array pitch is 50 nm larger than the design target due to the tolerance of our photolithography system.

## 4. Experimental Setup

**Figure 4:**
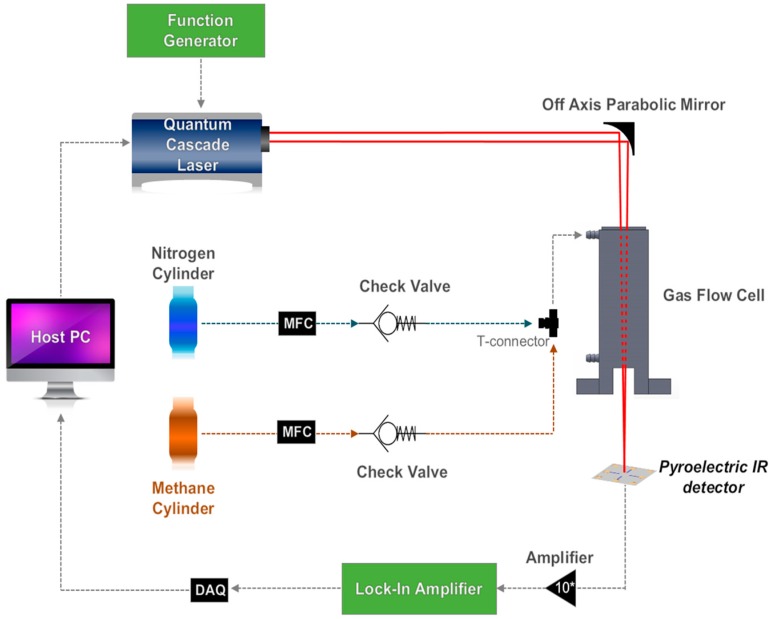
Schematic illustration of the experimental setup for NDIR spectroscopy.

The experimental setup used to characterize the pyroelectric IR detector is shown in Figure 4. The widely tunable quantum cascade laser (QCL), LaserTune^TM^ 3000 (Block Engineering, Marlborough, MA, USA) is used as a powerful IR source. 295 kHz pulsed output of 5% duty cycle is modulated at 25 Hz using the function generator 33500B (Keysight Technologies, Westlake Village, CA, USA) and focused onto the IR detector using an off-axis parabolic mirror. Before the IR laser beam reaches the IR detector, it passes through a 15 cm-long gas flow cell in which the spectrum and concentration of methane are to be measured. The output signal of the IR detector is first amplified 10 times using the operational amplifier AD524 (Analog Devices, Norwood, MA, USA) then filtered using the lock-in amplifier SR850 (Stanford Research Systems, Sunnyvale, CA, USA) at the modulation frequency of 25 Hz and recorded with a NI 6361 DAQ device (National Instruments Canada, Toronto, ON, Canada). Nitrogen and methane gas cylinders are used along with mass flow controllers (MFC) to supply methane to the gas flow cell at different concentrations. The wavenumber of the IR radiation is scanned from 1930 cm^−1^ to 930 cm^−1^ with a step size of 8 cm^−1^ (5.2 µm to 10.7 µm in wavelength).

## 5. Results and Discussion

**Figure 5:**
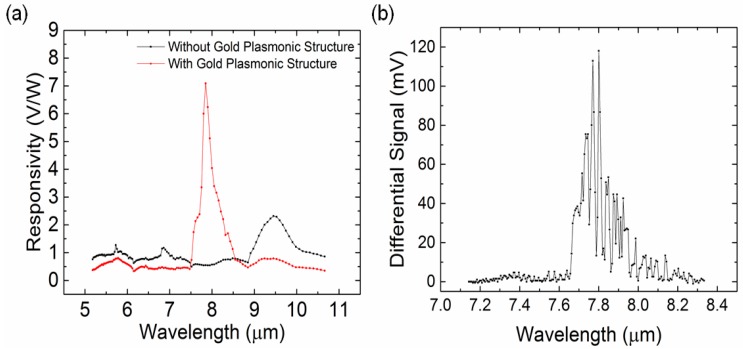
(**a**) The spectra of responsivities of the IR detector before (black) and after (red) adding the plasmonic crystal structure; (**b**) IR absorption spectrum of methane gas at a concentration of 30,000 ppm measured using the IR detector with the plasmonic crystal structure.

The responsivity of the IR detector is measured with the photoresist as the top layer before depositing the gold layer as well as after fabricating the gold plasmonic crystal structure without injecting any methane into the gas flow cell. The responsivity of the IR detector is defined as

(1) $$R=\frac{V_{\mathrm{out}}}{P_{\mathrm{in}}}$$

where $V_{\mathrm{out}}$ is the output signal of the IR detector before any amplification and $P_{\mathrm{in}}$ is the power of the IR radiation coming from the IR source. Note that $V_{\mathrm{out}}$ used for the evaluation of the responsivity of the IR detector is calculated from measured output signal divided by total amplification (100×) in our measurement setup. $P_{\mathrm{in}}$ is measured using the S401C thermal power head and PM100USB power and energy meter interface (Thorlabs Inc, Newton, NJ, USA). The spectra of the responsivity of the pyroelectric IR detector before and after adding the gold plasmonic crystal structure are shown in Figure 5a. Adding the gold layer leads to a maximum 13-fold enhancement in the responsivity of the IR detector at λ = 7.85 µm. The detection bandwidth of our measurement setup is 0.125 Hz and the noise floor level with our amplification circuit is measured to be 0.1 mV. Based on the definition provided in [30], the noise equivalent power (NEP) and the area-normalized detectivity of the IR detector are determined to be 4.04 × 10^−7^ W/$\sqrt{\mathrm{Hz}}$ and 1.2376 × 10^5^ $\mathrm{cm}\sqrt{\mathrm{Hz}}/W$, respectively. In addition, adding the plasmonic crystal structure reduces the responsivity of the IR detector at other wavelengths. This shows that the gold plasmonic crystal structure does function not only as an absorber, but also as an optical filter as reported in [3]. The IR absorption spectrum of methane at a concentration of 30,000 parts-per-million (ppm) (Figure 5b) is obtained using the fabricated IR detector in the range of 1400 cm^−1^ to 1200 cm^−1^ with a spectral resolution of 1 cm^−1^ (7.14 µm to 8.33 µm in wavelength) to show multiple absorption peaks in this spectral range. The differential signal in Figure 5b is defined as V_out,N2_-V_out,CH4_ where V_out,N2_ is the output signal of the IR detector when nitrogen gas is filled in the flow cell and V_out,CH4_ is the output signal of the IR detector when methane gas at specific concentration is filled in the flow cell, respectively. It should be noted that the sensor signal shown in Figure 5b is a result of the convolution of the absorptivity of methane and the responsivity of the IR detector. Thus, the maximum IR absorption peak is found to be at λ = 7.8 µm and the LOD of the NDIR methane gas sensing system is determined at this wavelength.

**Figure 6:**
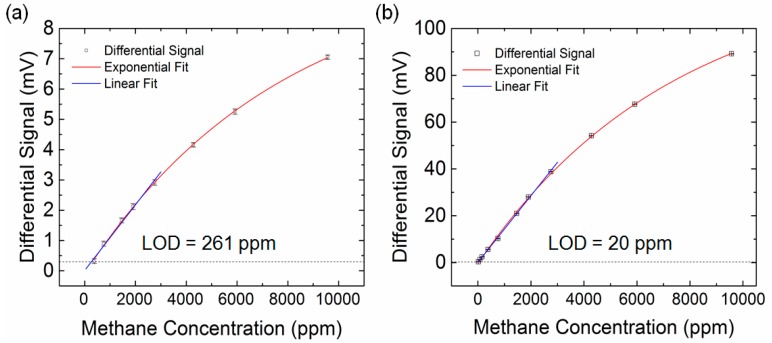
(**a**) The differential signals of the pyroelectric IR detector as a function of methane gas concentration before adding the plasmonic crystal structure. The red curve is an exponential fit of the differential signals and the blue curve is a linear fit of the differential signals. The LOD of the methane gas sensing system is determined by the intersection of straight line (blue) and the dashed line (black) at three times of the noise floor (0.3 mV); (**b**) The differential signals of the pyroelectric IR detector as a function of methane gas concentration after adding the plasmonic crystal structure. The red curve is an exponential fit of the differential signals and the blue curve is a linear fit of the differential signals. The LOD of the methane gas sensing system is determined by the intersection of straight line (blue) and the dashed line (black) at three times of the noise floor (0.3 mV).

The calibration curve of the NDIR gas sensing system is obtained by measuring the output signal of the IR detector at λ = 7.8 µm while varying the concentration of methane. The open squares in Figure 6 are averaged values of the differential IR detector signal at various methane concentrations with error bars showing the standard deviation of five measurements. The calibration curve of the NDIR gas sensing system before adding the gold plasmonic crystal structure is shown in Figure 6a. It is fitted by the exponential curve (red) which is in agreement with the Beer–Lambert law

(2) $$\Delta V=10.1\left(1-e^{-1.23\times 10^{-4}c}\right)$$

where Δ*V* is the differential signal, V_out,N2_-V_out,CH4_, in mV and c is the concentration of methane in ppm. For concentrations below 3000 ppm, the calibration curve can be approximated with a linear line (blue). The equation of the fitted line is Δ*V* = 0.01954 + 0.00108c with $R^{2}$ = 0.989. We define the LOD of the sensing system to be the concentration at which the SNR is 3. Based on this definition with our measured noise floor of 0.1 mV, the LOD is found to be 261 ppm. Figure 6b shows the calibration curve of the NDIR gas sensing system after adding the gold plasmonic crystal structure to the pyroelectric IR detector. It is fitted by the exponential curve (red) which is in agreement with Beer–Lambert law

(3) $$\Delta V=122.9\left(1-e^{-1.36\;\times \;10^{-4}c}\right)$$

This curve can also be approximated for concentrations below 3000 ppm with a linear line (blue). The equation of the fitted line is ΔV = 0.12907+0.01421c with R^2^ = 0.99. The LOD in this case is found to be 20 ppm. This demonstrates that adding the gold plasmonic crystal structure leads to overall 13-fold enhancement in the LOD of the NDIR methane gas sensing system. This is in line with the 13-fold enhancement in the responsivity of the IR detector shown in Figure 5a. Since our NDIR methane gas sensing signal follows Beer–Lambert law, we expect the LOD of our sensing system can be further improved by orders of magnitude by employing multipass cells with optical pass lengths from tens of meters to kilometers [23].

## 6. Conclusions

In conclusion, a gold plasmonic crystal structure is designed, optimized by numerical simulations, and fabricated on top of our PZT sensor [28] to demonstrate a strategy to enhance the responsivity of IR detectors. Integrating this into an NDIR methane gas sensing system achieves a 13-fold enhancement in the LOD. The design of the gold plasmonic structure we exploited in this paper can be easily implemented using conventional microfabrication techniques and is also not sensor specific. It can be implemented with any commercially available thermal IR detector.

## Figures and Tables

**Table 1:** The properties of materials used in the thermal simulation of the sensor.

Material	Density (kg/m^3^)	Thermal Conductivity (W/mK)	Specific Heat Capacity (J/kgK)
Gold	19,300	110	129
Photoresist	1000	0.4	1000
Platinum	21,440	50	136
PZT	7900	3.8	405
Silicon	2330	148	712
Silicon dioxide	2220	1.46	750
Silicon nitrate	2400	4	700

**Figure A1:**
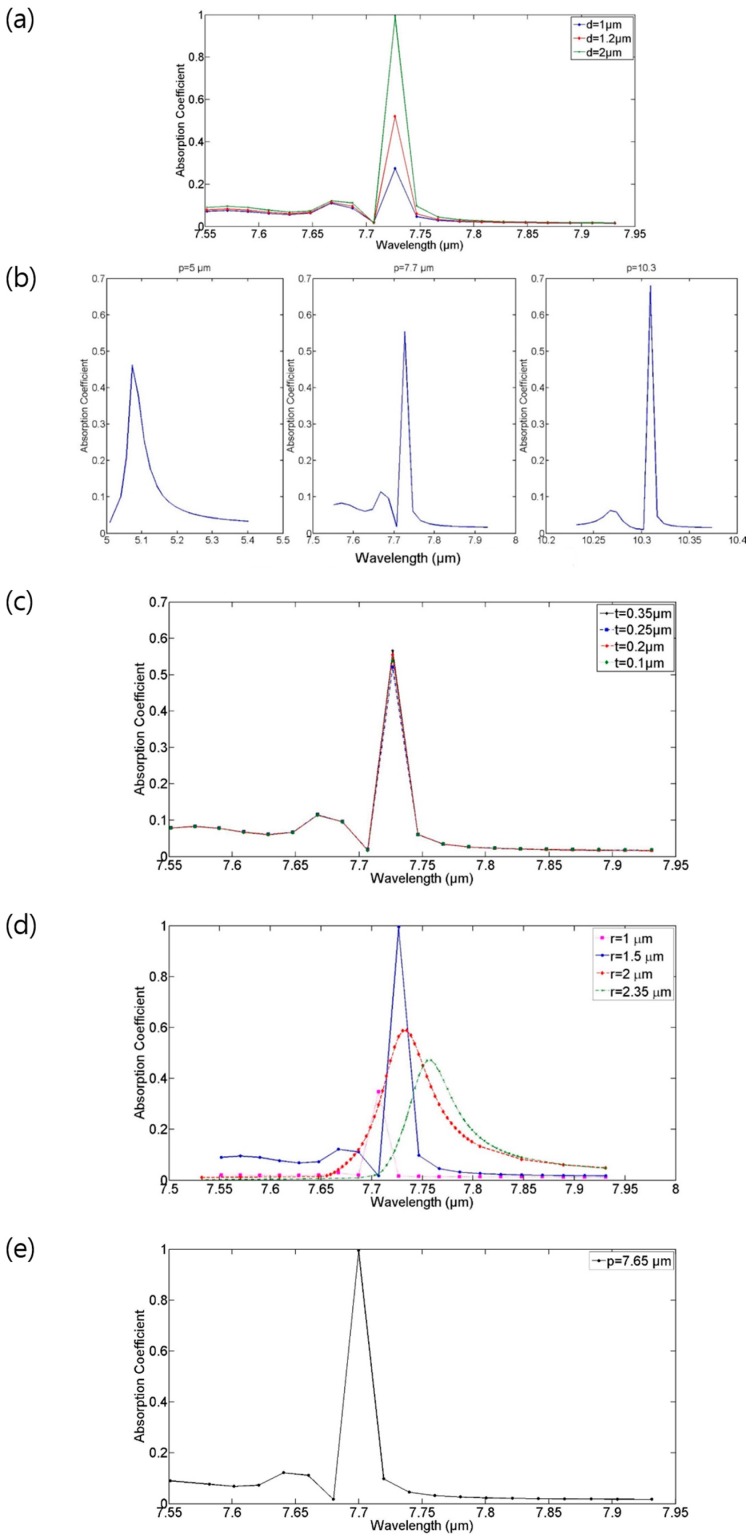
(**a**) The effect of the hole depth on the absorption spectrum; (**b**) The effect of the pitch on the absorption spectrum; (**c**) The effect of the gold layer thickness on the absorption spectrum; (**d**) The effect of the hole radius on the absorption spectrum; (**e**) Absorption spectrum of the final design of the plasmonic crystal structure: *p* = 7.65 µm, *r* = 1.5 µm, *d* = 2 µm, and *t* = 0.1 µm.

## Appendix A

To find the effect of the hole depth on the absorption spectrum, the numerical simulation is performed at the hole depths *d* = 1, 1.2, and 2 µm. Other dimensions of the plasmonic crystal structure are kept constant at *t* = 0.25 µm, *p* = 7.7 µm, and *r* = 1.5 µm. The results are shown in Figure A1a. It is clear from the figure that the depth does not affect the position of the absorption peak, however, it is clear that increasing the depth leads to enhancing the absorption at the desired wavelength. As the depth increases from 1 µm to 1.2 µm to 2 µm, the absorption increases from 27.2% to 52.1% to a near perfect absorption of 99.5%, respectively.

To investigate the effect of changing the pitch on the absorption spectrum, the numerical simulation is performed at three different pitches of *p* = 5, 7.7, and 10.3 µm. Other dimensions of the plasmonic crystal structure are kept constant at t=0.25 µm, d=1.2 µm, and r=1.5 µm. The results are shown in Figure A1b. It is clear from the figure that the wavelength of the absorption peak is substantially affected by the pitch. It is nearly equal to the pitch. At a pitch of 5 µm, 7.7 µm, and 10.3 µm, the absorption peak is located at 5.07, 7.727, and 10.31 µm, respectively.

To find the effect of gold layer thickness on the absorption spectrum, the numerical simulation is performed at four different thicknesses of t=0.1, 0.2, 0.25, and 0.35 µm. Other dimensions of the plasmonic crystal structure are kept constant at d=1.2 µm, p=7.7 µm, and r=1.5 µm. The results are shown in Figure A1c. It is clear from the figure that the thickness has a negligible effect on the absorption spectrum.

To investigate the effect of the radius of the hole on the absorption spectrum, the numerical simulation is performed at four different hole radius of r=1, 1.5, 2, and 2.35 µm. The results are shown in Figure A1d. It is clear from the figure that increasing the radius of the hole always leads to a red shift in the peak to a slightly longer wavelength. As the radius increases from 1 µm to 1.5 µm to 2 µm to 2.35 µm, the peak shifts from 7.07 µm to 7.727 µm to 7.735 µm to 7.759 µm, respectively. In addition to that, increasing the radius leads to an increase in the width of the peak. It should be noted that sharp peaks are preferred in our application because they make the plasmonic crystal structure to function as an optical filter. This suggests that *r* = 1.5 µm is an optimum dimension for our application.

Based on this parametric study, the optimal dimensions of the gold plasmonic structure are determined to be p = 7.65 µm, r = 1.5 µm, d = 2 µm, and t = 0.1 µm. The simulated absorption spectrum of the final design is shown in Figure A1e.
